# Non-linear optical measurement of the twist elastic constant in thermotropic and **DNA** lyotropic chiral nematics

**DOI:** 10.1038/s41598-017-05136-z

**Published:** 2017-07-10

**Authors:** Liana Lucchetti, Tommaso P. Fraccia, Fabrizio Ciciulla, Tommaso Bellini

**Affiliations:** 1 Department SIMAU, Università Politecnica delle Marche, Ancona, Italy; 2 Department Medical Biotechnology and Translational Medicine, Università di Milano, Milano, Italy; 3 Department Promotion of Human Sciences and Quality of Life, Università Telematica San Raffaele, Roma, Italy

## Abstract

Throughout the whole history of liquid crystals science, the balancing of intrinsic elasticity with coupling to external forces has been the key strategy for most application and investigation. While the coupling of the optical field to the nematic director is at the base of a wealth of thoroughly described optical effects, a significant variety of geometries and materials have not been considered yet. Here we show that by adopting a simple cell geometry and measuring the optically induced birefringence, we can readily extract the twist elastic coefficient K_22_ of thermotropic and lyotropic chiral nematics (N*). The value of K_22_ we obtain for chiral doped 5CB thermotropic N* well matches those reported in the literature. With this same strategy, we could determine for the first time K_22_ of the N* phase of concentrated aqueous solutions of DNA oligomers, bypassing the limitations that so far prevented measuring the elastic constants of this class of liquid crystalline materials. The present study also enlightens the significant nonlinear optical response of DNA liquid crystals.

## Introduction

Liquid crystals (LC) are a state of matter whose structured anisotropic and yet fluid nature has enabled an enormous variety of applications, most of which relying on the easy coupling of their symmetry axes to external fields. Because of their soft elasticity, LC readily respond to stimuli deforming their structures to balance external coupling with elastic restoring forces. For some class of systems this phenomenology has been thoroughly explored, such as for thermotropic nematics in electric and optical fields^1–8^. At the same time, the response to external fields of other systems and phases have been less explored. This is the case of the response of chiral nematic (N*) phases to optical fields – the object of this study – which has been studied primarily for their effects on the selective reflection of light having wavelength comparable to the chiral pitch^9–11^. This is also the case of water-based lyotropic LC, in which the water screening and ionic currents impair efficient coupling to electric fields. This limitation has been recently circumvented by the use of magnetic fields to determine the splay, bend and twist elastic constants of a lyotropic chromonic LC^12^. No previous data is instead available on the study of lyotropic LC by direct coupling with optical fields. Here we propose an all-optical method for the measurement of the twist elastic constant K_22_ in both thermotropic and DNA lyotropic chiral nematics (N*). The method is based on a conventional pump-probe set-up to measure light-induced optical birefringence. We have investigated cells in which the N* helical axis is perpendicular to the cell plates, a condition that in simple thermotropic LC is achieved by planar surface alignment. We describe here the application of this strategy to chiral-doped 4-cyano-4′-pentylbiphenyl (5 CB), a well-known and fully characterized thermotropic LC, doped by a chiral agent. The value of K_22_ we obtain for 5CB well matches those reported in literature, thus strengthening our confidence that this all-optical method could be extended to water-based lyotropic LC, in which the use of low-frequency electric fields is difficult or ineffective.

As a testbed for this study we selected a LC resulting from the self-assembly of oligomeric DNA, a class of lyotropic systems introduced and characterized in the last years^13–15^. In these systems the LC ordering is produced by the linear association of duplex-forming sequences, which in turn induces a macroscopic orientational ordering. Specifically, we investigated aqueous solutions of a “AT-DD” 14-base-long DNA having sequence 5′-ATCGCGAATTCGCG-3′. Since the underlined portion of this sequence is self-complementary (and is known as Dickerson Dodecamer “DD”), these molecules readily pair in stable duplexes as they are dissolved. The overhanging two-bases (AT) provide a hook to the AT overhang of other duplexes, favoring their chaining into weakly bound reversible polymers. In the AT-DD solutions, the N* phase is produced by the following staged self-assembly processes: oligomers couple by Watson-Crick pairing and form double-helix shaped duplexes; duplexes aggregate in linear chains because of AT mediated end-to-end interactions; chained duplexes develop orientational order along a common axis, defining the local nematic director. Because of the intrinsic chirality of the helical shaped DNA duplexes, the director spontaneously twists, yielding the typical periodic pattern of chiral nematic phases.

While the thermodynamics of DNA LC have been extensively characterized and modeled^13–16^, no data is so far available on their elastic properties. The hierarchical self-assembly at the basis of the LC ordering in oligomeric DNA make this system markedly different from thermotropic LC since in this case the appearance of LC phases involves not only the orientational ordering of the molecules, but also their degree of association^16^. At the same time, DNA LC are also different from standard chromonic LC – a class of self-assembling discotic lyotropics^12^, since the aggregating unit has a variable size, determined by the length of the oligomers. It would be therefore of interest being able to access the elastic properties of DNA LC, which are expected to depend at the same time on the interduplex interaction strength, on the duplex flexibility and on the flexibility of the aggregated structure.

However, measuring elasticity in DNA LC has turned out to be cumbersome because of a combination of difficulties: DNA LC are intrinsically chiral, making it difficult to use typical light scattering based analysis; no strategy has emerged yet to control the surface alignment of DNA LC phases, ruling out the possibility of using Freedericksz transition methods; coupling with electric fields is weak and disturbed by the large density of intrinsic counterions present in the solution. All these limitations are here overcome by using an optical field and by exploiting a geometry in which surface alignment is not crucial.

**Figure 1:**
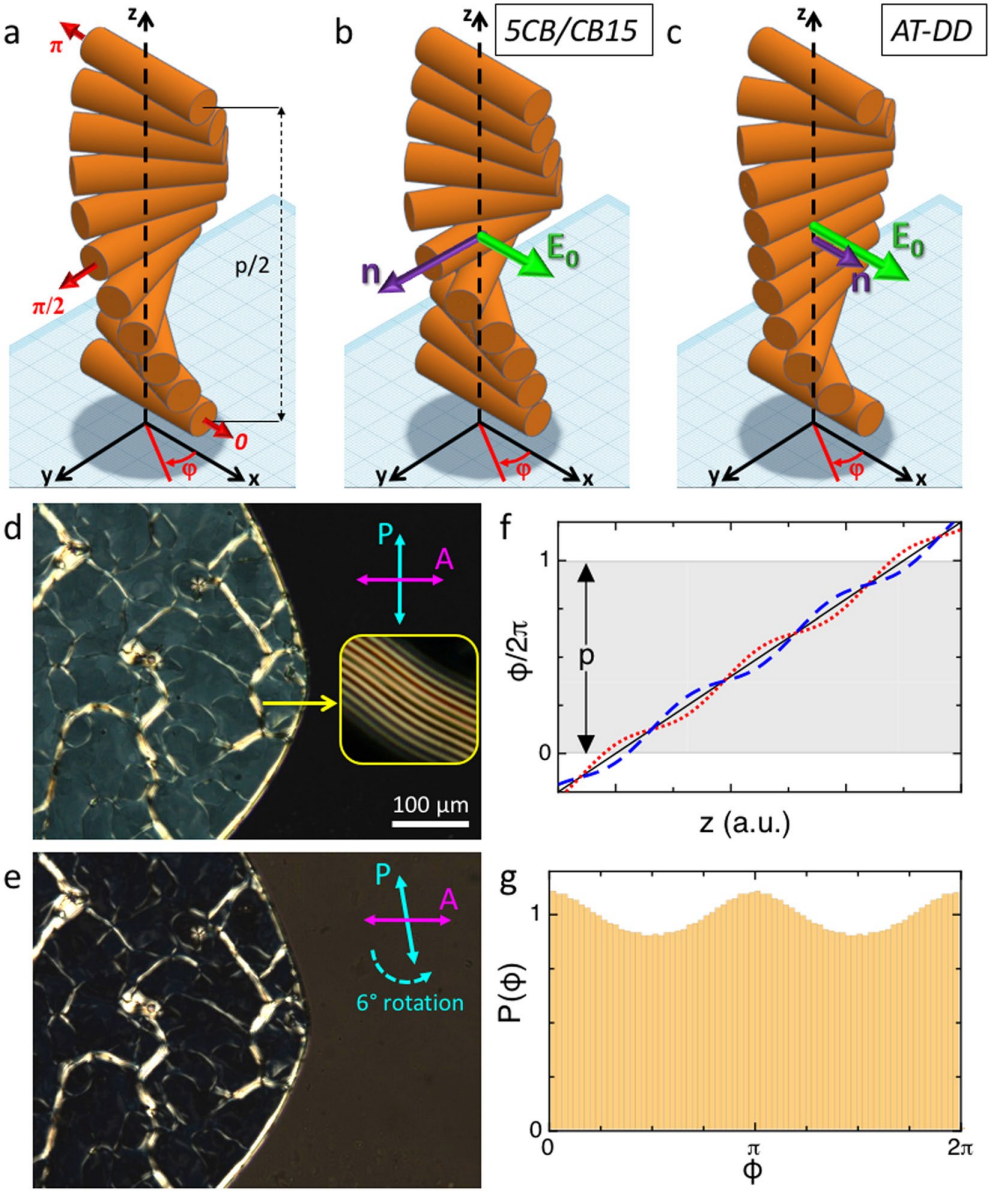
Description of the cell geometry and of the effects of the optical coupling. (**a**–**c**) Sketches of the z-dependence of the optical axis, here represented by the orientation of cylinders, in the three cases of: unperturbed planar (left handed) N* cell (**a**), coupling with the optical field in case of a N* phase with positive dielectric anisotropy such as the thermotropic LC 5CB/CB15 favoring alignment of the director along the field (**b**), coupling with the optical field in case of a N* phase with negative dielectric anisotropy such as the lyotropic AT-DD solutions favoring alignment of the director normal to the field (**c**). The drawing extends on half of a pitch. φ is the angle of the local director with the x axis. (**d**,**e**) Images in polarized transmission optical microscopy of a AT-DD cell through crossed polarizers (**d**) and through polarizers uncrossed in such a way to minimize the intensity transmitted by the DNA solution (**e**). This behavior demonstrates optical rotation. Uniform planar domains are surrounded by whitish-appearing oily streak textures. Inset in (**d**) shows an oily streak pattern at higher magnification displaying fingerprint texture. (**f**) z-dependence of φ in the three cases on panels a–c: unperturbed call (continuous black line), Δε > 0 (dotted red line), Δε < 0 (dashed blue line). Perturbations as in Eq. 2. (**g**) Histogram distribution of the optical axis orientation φ in the x-y plane for a N* coupled with positive dielectric anisotropy to an optical field polarized along x. P(φ) is obtained from the profile in Eq. 2 when z is limited to an integer number of pitches.

We report here experiments performed on N* cells in planar geometry, where the chiral axis of cholesterics develops along z, the direction normal to the cell surfaces. Figure 1a offers a pictorial description of the progression of the optical axis along z. In the case of 5CB/CB15 the planar geometry is obtained by controlling the surface coupling by rubbing, while in AT-DD this geometry is spontaneously attained within large domains in the sample. 5CB/CB15 and AT-DD cells have been irradiated by a linearly polarized pump beam at normal incidence. Since the average size of the AT-DD domains was larger than the width of the probe beam, even in the defected DNA system we could perform experiments in uniform regions. The coupling with the optical field produces a change in the director orientation by favoring alignment in the direction of the field or perpendicularly to it depending on the sign of the dielectric anisotropy Δε. The perturbation in the optical axis produced by this coupling is sketched in Fig. 1b and c for the case of 5CB/CB15 (Δε > 0) and AT-DD (Δε < 0), respectively. This perturbation, whose amplitude results from the balance of the coupling to the optical field and the twist elastic restoring force, corresponds to the appearance of an induced birefringence in the LC cells with axis parallel to the optical field, which we measured by a probe beam.

The results here reported highlight the remarkable nonlinear optical response of liquid crystalline DNA, which, to the best of our knowledge was never observed before. Several papers have been published in the last decade reporting the nonlinear optical response of functionalized DNA in isotropic solutions in view of possible applications in the field of photonics^17, 18^. Because of the collective molecular ordering, the optical nonlinearity of liquid crystalline DNA is much larger than in isotropic solutions, an effect to be considered within such investigations of DNA in the context of photonics and biophotonics.

## Methods

Thin planar cells have been prepared for the two systems here considered: thermotropic N* cells made by 5CB doped with a small amount of the chiral dopant CB15 and DNA lyotropic N* cells.

Thermotropic LC cells have been prepared by assembling two PVA coated glasses, rubbed to get planar alignment of the molecular director. Cell thickness d, as determined by mylar spacers, is d = 50 μm. A mixture of 5CB and the chiral dopant CB15, has been used to fill the cells. Dopant weight concentration of 4–6% allowed obtaining chiral nematic samples with pitch p ≈ 2.9 μm and p ≈ 1.7 µm, as measured from the fingerprints pattern transitorily appearing after temperature quenching of the cell (as described later on). The good planar alignment of the samples has been checked by polarizing optical microscopy. Due to the planar orientation of the chiral nematic, the helix axis is orthogonal to the two cell substrates, as sketched in Fig. 1a.

AT-DD cells were prepared by depositing droplets of the DNA solution in MilliQ water on a clean untreated glass substrate, letting them concentrate by evaporation, closing the cell with a second glass plate held about 20 µm apart by silica spacer and sealing the cell by fluorinated oil. Gaps in all cells and DNA concentration (c_DNA_) were measured after equilibration by microscope-based interferometry^13^. Data shown in this paper are obtained from samples with c_DNA_ ≈ 550 mg/ml, a figure to be compared with the density of 1800 mg/ml of lyophilized DNA. At this concentration, the solution forms a chiral nematic phase. As mentioned, DNA nematics cannot be easily aligned by surface treatment. However, when cells are thin enough, the N* phase spontaneously aligns with large (about 100–400 µm diameter) domains having the helical axis normal to the cell planes. This is shown in Fig. 1d reporting a picture of the AT-DD cell observed in transmission light microscopy through crossed polarizers. The texture is typical for the N* phase. The grey tiles are domains in which the cholesteric axis is along z, and the whitish “oily streaks” are narrow regions in which the N* helix is in the plane of the cell. When observed at higher magnification, these regions might show “fingerprint” patterns, such as those in the inset of Fig. 1d, from which it is straightforward to determine p as twice the distance between lines^19^. In the cells considered in the experiments here reported p has been found to be in the range 3.5–5 µm. Data analyzed and discussed below have been taken in a cell with p = (4.15 ± 0.05) µm. The local birefringence is about Δn_0_ ≈ 0.02, as obtained from c_DNA_ as described elsewhere^13^. Since nucleobases lay perpendicular to the nematic director, both birefringence and dielectric anisotropy are negative.

**Figure 2:**
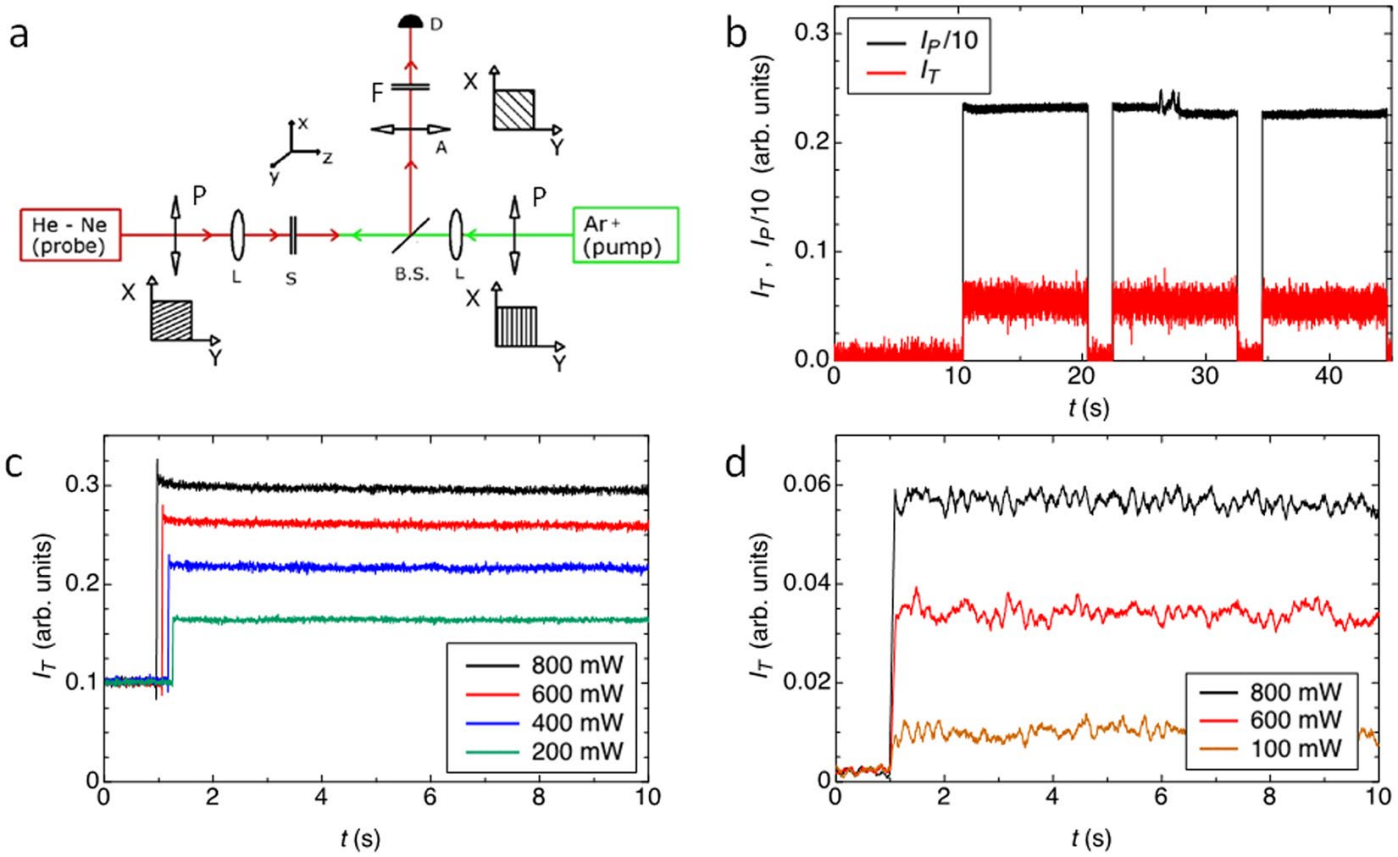
Experimental setup and transmitted intensity data. (**a**) Sketch of the experimental apparatus. The different optical components are indicated: P = polarizers, L = lens, S = sample, B.S. = beam splitter, A = analyzer, D = detector, F = filter. A pictorial description of the optical field direction is shown for each polarizer/analyzer. (**b**) Typical example of the observed response in terms of transmitted intensity (red line) to the pump optical field (black line) as the pump beam in turned on and off in time. The case of a AT-DD cell is reported in the case of polarizers set to the extinction orientation.Sampling time 4 ms. (**c**,**d**) Transmitted probe signal for different values of the pump power as a function of time, for the thermotropic 5CB/CB15 (**c**) and for the lyotropic AT-DD (**d**) chiral cells. The pump beam polarization is parallel to the x axis. Data averaged on 40 ms.

Measurements were performed with a pump-probe set up sketched in Fig. 2a. The pump field is provided by an Ar^+^ laser (λ = 514 nm) that illuminates the samples at normal incidence. The incident light is linearly polarized either along x or y (that is either parallel or perpendicular to the rubbing direction in thermotropic cells) and is focused by a 10 cm plano convex lens. This gives a beam diameter on the sample of 50 μm, smaller than the average size of the planar domains in DNA cells. Pump power ranges from 100 to 800 mW. A mechanical shutter enabled performing irradiation cycles, which in the experiments here reported had 10 s duration and 2 s dark time separation.

The nonlinear optical response of the samples has been studied by means of a low power counter-propagating probe beam (λ = 633 nm) also normally incident and focused on the sample at the center of the pump spot. The probe light transmitted by the cell was sent through an analyzer and monitored by a photodiode connected to a PC. A series of filters prevent the pump light to reach the detector. The polarization of the probe light is chosen so to form an angle of 45° with respect to that of the pump beam. This requires a little attention since, because of the chiral arrangement of the director in the N* phase, both 5CB/CB15 and DNA cells generate optical rotation. This is shown in Fig. 1d and e, where it is apparent that the condition of crossed polarizers (Fig. 1d) is not the one in which the transmission through the domains is minimized. Near extinction is instead obtained by rotating the analyzer counter-clockwise of about 6° (while in 5CB/CB15 – a system with stronger birefringence – extinction is obtained with a rotation of the order of 20°). The pictures in Fig. 1d and e demonstrate that this system produces clockwise optical rotation in the linear polarization as light travels across the cell, in turn indicating left-handed N* symmetry, in agreement with previous observations^20–22^.

In the presence of optical rotation, the condition of 45° between pump and probe beams can be achieved by holding the analyzer of the probe beam at 45° with respect to the incident polarization of the pump, while rotating the polarizer of the probe to minimize the probe light transmitted by the analyzer. In other words, since probe and pump polarizations rotate approximately by the same angle when crossing the sample, if their relative angle is set at 45° on the face of the sample where the pump enters the cell (and where the probe exits the cell), the same angle is maintained throughout the whole cell. Therefore, when the sample is investigated at extinction, the outcome of the measurements is the same as if no optical rotation were present.

Measurements were performed either at room temperature (T ≈ 25 °C, held by the thermostat of the laboratory), or at variable T, as controlled by a CaLCTech hot stage.

## Results

The typical cell response to the pump optical field can be observed in Fig. 2b, where both the transmitted probe (red) and the pump (black) signals are shown as a function of time for the choice of polarizers and analyzer orientations described above, in the case of DNA samples. As it can be seen the response time is faster than the sampling time τ (τ = 4 ms) and it cannot be thus analyzed with the current experimental setting. It will be measured and discussed in the future to determine the rotational viscosity of DNA N* phases.

**Figure 3:**
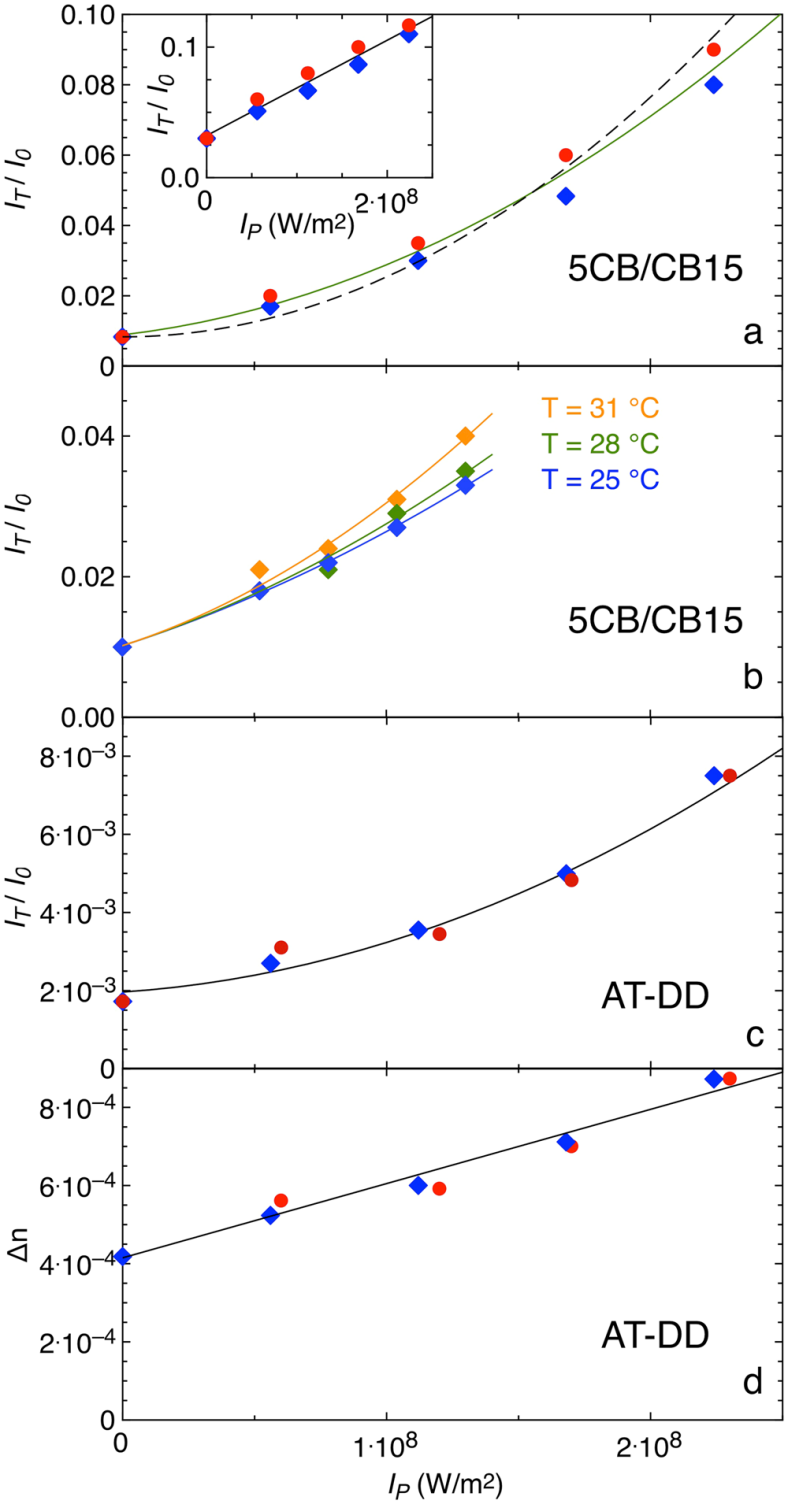
Optically modulated transmission and induced birefringence. (**a**,**b**,**c**) Transmitted probe beam intensity (*I*_*T*_) normalized to the incident probe intensity (*I*_*0*_) as a function of the pump intensity (*I*_*P*_) polarized along x (blue diamonds) and along y (red dots) measured in 5CB/CB15 at room T (**a**), in 5CB/CB15 at three different T (**b**), and in the AT-DD cell (**c**). Measurements are performed with polarizers at extinction. Dashed lines: fit to the data with Eq. 7. Continuous lines: fit to the data with Eq. 9. Inset: same measurements with analyzer rotated away from the extinction position, fitted with a linear dependence (continuous line). (**d**) Optically induced birefringence in the AT-DD N* phase extracted from the data in panel c by Eq. 1 fitted with a linear dependence (line).

In Fig. 2c and d we show the transmitted probe signal for different values of the pump power, for both systems, and with pump beam polarization along x. From these and analogous data we determined the amplitude of *I*_T_, the light transmitted by the analyzer, normalized to *I*_0_, the probe beam intensity, as a function of *I*_P_, the pump intensity. The results are shown in Fig. 3a,b (5CB/CB15) and 3c (DNA) for both pump beam polarizations (i.e along x and y).

For comparison, and to evaluate the possible relevance of thermal effects due to heating by pump light absorption, we performed analogous experiments with circularly polarized pump beam. We could observe no effect in both systems, which rules out this possibility. This is also consistent with the relatively fast switching time and with the absence of drifts in *I*_T_/*I*_0_ during the application of the pump field.

The intensity transmitted through crossed polarizers sandwiching a layer of thickness d and birefringence Δn oriented at 45° with respect to the polarizers is^23^:

1 $${I}_{T}={I}_{0}\,\sin \,{(\frac{\delta }{2})}^{2}$$

where δ is the phase shift induced by the birefringence: $\delta =\frac{2\pi }{\lambda }d{\Delta }n$. Thus, by interpreting the signal as an optical Kerr effect^24^, we expect for small induced birefringence *I*_*T*_/*I*_0_ ∝ Δ*n* ^2^ and thus, at the lowest order, ${I}_{T}/{I}_{0}\propto {I}_{P}^{2}$.

## Discussion

In a planar N* cell, the unperturbed optical axis **n**(z) forms a uniform spiral, with the angle φ formed with the x-axis linearly growing with z as *φ* = (2*π*/*p*)*z*, as drawn in Fig. 1f (continuous line). Thus, in a cell with an integer or semi-integer number of pitches, such as the one containing the mixture 5CB/CB15, all the optical axis is uniformly distributed in the x-y plane, i.e. the angular distribution P(φ) is constant, corresponding to no effective birefringence experienced by light travelling along z.

As the system is exposed to a linearly polarized beam having the optical field in the x-y plane, an optical torque is exerted on **n** favoring its alignment either with or perpendicularly to the field. This is sketched in Fig. 1b and c, where we assume that the optical field is aligned with the x-axis, and that the dielectric anisotropy Δε is positive, as in the case of 5CB, or negative, as in the case of AT-DD N*, respectively. Accordingly, φ(z) is perturbed by the optical field to favor an increase in P(φ) along (or perpendicularly) to the polarization direction of the pump. For weak optical coupling, we can assume a sinusoidal perturbation:

2 $$\phi =\frac{2\pi }{p}z+A\,\cos \,\frac{4\pi z}{p}$$

where A is the amplitude of the perturbation. In Fig. 1f we plot Eq. 2 for A = 0, 0.1, −0.1. The resulting P(φ) evaluated for a z interval corresponding to one pitch is plotted in Fig. 1g for A = 0.05. The order parameter resulting from the angular distribution in Eq. 2 is *P*_2*XY*_ = *A* = Δ*n*_*eff*_/Δ*n*_0_, where Δn_eff_ is the birefringence experienced by light travelling along z, while Δn_0_ is the local intrinsic birefringence of the N* phase.

The amplitude A in Eq. 2 can be computed from the competition between the coupling to the optical field and the elastic response. Specifically, by calling K_22_ and k_T_ the twist elastic coefficient and the chiral torque, respectively, we obtain that the elastic free energy density *f*_E_ is:

3 $${f}_{E}=\frac{1}{2}{K}_{22}\frac{{\partial }^{2}\phi }{\partial {z}^{2}}-{k}_{T}\frac{\partial \phi }{\partial z}=\frac{2{\pi }^{2}{K}_{22}}{{p}^{2}}(1+2{A}^{2})+\,\frac{2\pi {k}_{T}}{p}$$

When the sample is unperturbed, minimization leads to A = 0 and *p* = 2*πK*_22_/*k*_*T*_, as expected^25^. In the presence of an optical field, **n** couples to the polarization direction through Δε yielding a non-zero free energy density *f*_0_:

4 $${f}_{O}=(1+A)\,\frac{{\rm{\Delta }}{\epsilon }{E}^{2}}{32\pi }$$

where E is the amplitude of the pump optical field.

By minimizing *f*_E_ + *f*_O_ with respect to A we find:

5 $$A=\frac{{\rm{\Delta }}{\epsilon }{E}^{2}{p}^{2}}{256{\pi }^{2}{K}_{22}}$$

and thus:

6 $${\rm{\Delta }}{n}_{eff}=\frac{{\rm{\Delta }}{n}_{0}^{2}{p}^{2}{I}_{P}}{16\pi c{K}_{22}}.$$

The presence of the optical field could also in principle modify the value of p^26, 27^. We evaluate this effect by minimizing *f*_E_ + *f*_O_ with respect to p. We obtain that the pitch in the presence of an optical field becomes *p*_*E*_ ≈ *p*(1 + 2*A* ^2^), i.e. a correction of a higher order which could be safely neglected since in the observations reported here *A* < 10^−2^.

An expression analogous to Eq. 6 for the optically induced birefringence in chiral nematics has been predicted several years ago by Zeldovich and Tabyrian^28^ by considering lattice-like perturbations of the director distribution. By considering a light beam propagating along the helix axis, they argued that the dominant effect was in the director perturbation within each pitch period, in agreement with our conclusions.

In Eq. 5 the parameter *A*, and thus the birefringence induced by the pump laser light, depends on the optical field squared, coherently with the centrosymmetric nature of the N* ordering. In the absence of additional effects, we thus expect the transmitted probe light I_T_ (Eq. 1) to depend on I_P_ as:

7 $$\frac{{I}_{T}}{{I}_{0}}=B{I}_{P}^{2}$$

with

8 $$B=\frac{{\delta }^{2}}{4}\frac{1}{{I}_{P}}=\frac{{\rm{\Delta }}{n}_{0}^{4}{d}^{2}{p}^{4}}{256{c}^{2}{\lambda }^{2}{K}_{22}^{2}}.$$

In practice Eq. 7 does not fully capture the observed behavior.

As previously mentioned, as probe light travels across the cells, its polarization rotates. This effect of optical rotation, expected when the optical wavelength is shorter than p, maintains the linear polarization of the incident light in the limit pΔn ≪ λ, a condition satisfied by AT-DD but not by 5CB/CB15 samples. In the latter, we thus expect that the probe polarization not only rotates but also turns from linear to slightly elliptical. Therefore, the light selected by the analyzer when oriented to minimize transmission is not fully extinguished. Indeed, the value of *I*_T_/*I*_0_ > 0 for *I*_P_ = 0 in Fig. 3a is larger than background due to stray light and optical imperfections, which we independently measured. This is also confirmed by the poor quality of a fit to the data based in Eq. 8 plus an adjustable constant accounting for stray light (dashed line in Fig. 3a).

On this basis, we interpreted the probe light transmitted by the analyzer in 5CB/CB15 cells as the sum of two contributions, one resulting from non-ideal optical rotation and the other due to the birefringence induced by the pump light. The two fields, having a fixed phase difference, mix giving rise to an additional contribution to *I*_T_ that linearly depends on *I*_P_^29^. Eq. 7 has thus to be modified as:

9 $$\frac{{I}_{T}}{{I}_{0}}=B{I}_{P}^{2}+C{I}_{P}$$

Because of the different dependence on *I*_P_ of the two terms, data fitting enables determining both B and C. In the case of 5CB/CB15, such fit, shown in Fig. 3a as a continuous line, yields B ≈ 8.5 10^−19^ m^4^/W^2^. Since all the other quantities in Eq. 8 are known, the fitting procedure enables determining the twist elastic constant at room temperature to be K_22 _≈ 2.7 10^−12^ N, in full agreement with the values reported in literature^30–33^. For comparison, should we have remained to the concept of *I*_P_ = 0 transmission as background light and thus not have included the linear term in Eq. 9, we would have obtained K_22_ ≈ 1.9 10^−12^ N (dashed line in the Fig. 3a), a value smaller but close to the previous. The relevance of the mixing condition expressed in Eq. 9 appears clearly when the polarizer is rotated away from the extinction position. In this case, the background is larger and the linear component in Eq. 9 grows to the point of dominating the signal, as shown in the inset of Fig. 3a.

As a further test of the adequacy of the method to determine the twist elastic constant, we explored how the non-linear optical response of 5CB/CB15 depends on T. This is shown in Fig. 3b, where *I*_T_/*I*_0_ vs. *I*_P_ is shown for three temperatures. The analysis, performed as above using Eq. 9 (lines represent best fits) yields K_22_ ≈ 3.1 10^−12^ N (for T = 25 °C), K_22_ ≈ 2.5 10^−12^ N (for T = 28 °C), K_22_ ≈ 1.6 10^−12^ N (for T = 31 °C), again in agreement with the expected weakening of the elastic constant upon approaching the transition T^30–33^. These evaluations were performed by using 5CB birefringence data as published in ref. 34.

*I*_T_/*I*_0_ vs. *I*_P_ for AT-DD is shown in Fig. 3c. Here again, data do not match the idealized condition of Eq. 7. Due to the much lower birefringence of AT-DD, the propagation of light through these samples should be simpler to describe than in the case of 5CB/CB15 since a pure rotation of the linear polarization is expected and thus the mixing term considered before should be negligible. However, the interpretation of AT-DD behavior is not straightforward because DNA cannot be easily coupled to the cell surfaces. Indeed, DNA LC samples appears to be free to align in directions that do not depend on the surfaces but rather on their thermal history and on the minimization of their bulk free energy. Therefore, missing an easy axis on the surfaces, the number of pitches across the cell is not constrained to be integer or half-integer, as in the case of 5CB/CB15. This has the relevant consequence that in general, even for *I*_P_ = 0, the birefringence of the cell is not zero. Its axis, however, is free to slide, since the coupling of the DNA with the surfaces is very weak. Therefore, the optical field can easily rotate the N* ordering around z and align the residual birefringence so to be perpendicular to its polarization (because of Δε < 0). We thus analyzed the data in Fig. 3c by assuming that the all transmitted intensity, even the one measured for *I*_P_ = 0, is due to birefringence aligned by the pump light. We thus used Eq. 1 to extract δ and Δn from the experimental data of Fig. 3c. The results are shown in Fig. 3d. Δn(*I*_p_) appears to be given by a constant term plus a term linearly depending on *I*_p_, a behavior suggesting that our interpretation is correct.

By virtue of Eq. 6, the slope of Δn vs *I*_p_ enables determining the twist elastic constant for AT-DD at room temperature. We found K_22_ ≈ 2.5 10^−13^ N. This value does not have a direct reference of comparison, since no elastic coefficient of DNA LC were previously determined. However, it appears to be in the same order of magnitude with the twist elastic constant determined in the nematic phase of chromonics, a class of lyotropic where ordering is the result of the stacking into columns of amphiphilic flat molecules^12, 35^. In these self-assembled achiral system, it has been found that K_22_ is in the range 0.25–1.75 pN, depending on concentration and temperature. Our result is thus at the lowest limit of their range. Another possible comparison is the approximate estimate obtained by modeling the columnar aggregates as polydisperse cylinders having a length distribution matching the one expected by the assembly of DNA oligomeric duplexes^22^. From that model, in conditions close to the one considered here, K_22_ ≈ 10^−12^ N. Here again our results are smaller but compatible with the model, in which various parameters could be adjusted, such as the flexibility of the aggregates. Moreover, the complex geometry of the DNA helices might bring about some additional complexities in their interactions that are not currently captured by the model. Overall the low value that we find for K_22_ still needs to be fully interpreted and might be a useful indication of structural features which are specific to DNA self-assembly, such as unexpectedly large flexibility or neglected peculiarities of DNA-DNA interactions.

The phenomenon here discussed can be described as a manifestation of the optical Kerr effect. Accordingly, the induced birefringence can be expressed in term of the non-linear coefficient n_2_ as ${\rm{\Delta }}{n}_{eff}={n}_{2}{I}_{P}$. LC are in general known to exhibit the so called Giant Optical Nonlinearity (GON), which results from the collective nature of their light-induced reorientation^36^. Typical values of the nonlinear coefficient for thermotropic LC in the nematic phase are on the order of n_2_ ≈ 10^−5^ cm^2^/W. The value we obtain from our experiments is much lower, n_2_ ≈ 10^−8^ cm^2^/W for both DNA LC and for 5CB/CB15. These small values of n_2_ can be understood on the basis of the different geometry adopted in our study. While in typical experiments on nematics the relevant length scale is the cell thickness, here director perturbation takes place within a pitch length. Indeed, in our geometry, the induced birefringence would be the same even in a cell only one pitch thick. We argue that the difference in n_2_ is a simple effect of the different length scales. In the case of weak director reorientation, the nonlinear coefficient in nematics is proportional to the cell thickness squared d^2^ ^37^. We should thus compare typical cell thickness (of the order of tens of microns) with lengths corresponding to a fraction of p, in which the director rotates for example by π/2 (i.e. in p/4). This corresponds, for the 5CB/CB15 mixture, to a length <1 µm. The squared ratio between the two lengths is enough to satisfactorily account for the 10^3^ difference between the n_2_ observed in our experiment and those reported in the literature for the GON. Worthy of note, the absence of GON in chiral nematics was theoretically predicted by Zeldovich and Tabyrian^28^.

It is also interesting to compare the nonlinear optical coefficient obtained here for DNA LC phase with those reported in the literature for this widely studied biopolymer. The largest values have been obtained very recently by investigating DNA in thin solid films^38^. The values reported for n_2_ are in the range 10^−13^–10^−11^ cm^2^/W, that is at least three orders of magnitude smaller than the one we report here. Indeed, the combination of cooperative DNA alignment and fluidity typical of LC ordering enable enlightening the optical properties of DNA, a feature that could further promote considering DNA in photonics and biophotonics applications.

## Conclusions

We describe an all-optical method for the measurement of the twist elastic constant K_22_ in both thermotropic and lyotropic chiral nematics. We have shown that this method, based on a simple pump-probe geometry, allows successfully extracting the twist coefficient K_22_ for chiral doped 5CB. We have also shown that the same method can be successfully applied to DNA chiral nematics, bypassing two main limitations that so far prevented the measurement of elasticity of DNA LC: the counterion screening that precludes the use of low-frequency electric fields, and the impossibility of controlling the surface alignment of DNA. The latter is overcome by working on single domains and by the fact that the characteristic length for the elastic response here considered is the N* pitch rather than the cell thickness. We obtain values of K_22_ in the range of a few 10^−13^ N, lower but comparable to those measured in chromonic liquid crystals.

Our experiments also highlight the remarkable existence of a easily detectable nonlinear optical response of DNA solutions. This effect, much larger than the one observed in the isotropic solutions of DNA, could represent a further incentive for the current investigation of potential applications of DNA in biophotonics.

This study opens the way to new measurements of the elastic and viscous response of lyotropic LC, some of which are currently under way.
